# The Death Effector Domains of Caspase-8 Induce Terminal Differentiation

**DOI:** 10.1371/journal.pone.0007879

**Published:** 2009-11-18

**Authors:** Ainhoa Mielgo, Vicente A. Torres, Michael C. Schmid, Ryon Graf, Samantha G. Zeitlin, Pedro Lee, David J. Shields, Simone Barbero, Colin Jamora, Dwayne G. Stupack

**Affiliations:** 1 Department of Pathology, School of Medicine, University of California San Diego, La Jolla, California, United States of America; 2 Moores Comprehensive Cancer Center, University of California San Diego, La Jolla, California, United States of America; 3 Department of Cellular and Molecular Medicine, School of Medicine, University of California San Diego, La Jolla, California, United States of America; 4 Division of Biological Sciences, University of California San Diego, La Jolla, California, United States of America; Roswell Park Cancer Institute, United States of America

## Abstract

The differentiation and senescence programs of metazoans play key roles in regulating normal development and preventing aberrant cell proliferation, such as cancer. These programs are intimately associated with both the mitotic and apoptotic pathways. Caspase-8 is an apical apoptotic initiator that has recently been appreciated to coordinate non-apoptotic roles in the cell. Most of these functions are attributed to the catalytic domain, however, the amino-terminal death effector domains (DED)s, which belong to the death domain superfamily of proteins, can also play key roles during development. Here we describe a novel role for caspase-8 DEDs in regulating cell differentiation and senescence. Caspase-8 DEDs accumulate during terminal differentiation and senescence of epithelial, endothelial and myeloid cells; genetic deletion or shRNA suppression of caspase-8 disrupts cell differentiation, while re-expression of DEDs rescues this phenotype. Among caspase-8 deficient neuroblastoma cells, DED expression attenuated tumor growth *in vivo* and proliferation *in vitro* via disruption of mitosis and cytokinesis, resulting in upregulation of p53 and induction of differentiation markers. These events occur independent of caspase-8 catalytic activity, but require a critical lysine (K156) in a microtubule-binding motif in the second DED domain. The results demonstrate a new function for the DEDs of caspase-8, and describe an unexpected mechanism that contributes to cell differentiation and senescence.

## Introduction

Caspase-8 is an initiator protease recruited to the death inducing signaling complex during apoptosis initiated by death receptors. Homotypic interactions, mediated by the amino-terminal death effector domains (DEDs) of caspase-8, are required for recruitment and subsequent maturation and dimerization of caspase-8 initiating the extrinsic apoptosis cascade. In addition to this role in death receptor-mediated apoptosis, cumulative evidence suggests that caspase-8 performs other non-apoptotic functions in development [1], including proliferation [2], [3], cell migration [4]–[7] and differentiation [3], [8]. We and others previously reported that caspase-8 has the capacity to localize to a number of different cellular locations, including the cytosolic compartment [9], actin-rich ruffles [10], [11], endosomes [12], including those at the front of migrating cells[13], focal adhesions [4] and stable microtubule structures, such as centrosomes [14]. Interestingly, the different domains of caspase-8 appear to favor localization to different cellular compartments. It is possible that these different preferred locations may ultimately influence caspase-8 function(s).

Differentiation, senescence, and apoptosis are critical programs for the development and maintenance of cellular homeostasis. Disruption of any of these essential processes is an important component in the pathogenesis of many diseases, including cancer. A common characteristic of human cancer is disrupted cellular differentiation [15]. In some cancer cells, the induction of differentiation with therapeutic agents terminates uncontrolled proliferation [16], [17]. Even among therapies which do not aim to specifically induce cell differentiation or senescence, it nonetheless appears to be a common mechanism limiting tumor growth [18]. Therefore, cellular senescence is not only a physiological program that limits the proliferative capacity of an indiviudal cell, but plays an important physiological role as a natural barrier to suppress pathogenic transformation or proliferation [19].

In the current study we observed in epithelial, endothelial, myeloid and tumor cells, that lack of caspase-8 exhibited a disrupted pattern of cellular differentiation/senescence. Specifically, we find that caspase-8 expressing myeloid, endothelial or epithelial cells display an accumulation of caspase-8 DEDs during the onset of differentiation and senescence. Silencing of caspase-8 correlates with disruption of differentiation, and increase in replicative capacity, while expression of the DEDs is sufficient to promote differentiation and proliferative arrest. In particular, these events occur independent of caspase catalytic activity, but require the ability of DEDs to bind to microtubules. The microtubule interaction capacity of caspase-8 DEDs, in turn, promotes aberrant mitosis, multinucleation, and induces apoptosis, senescence or terminal differentiation. Our results support the notion that caspase-8 is a multifunctional tumor suppressor protein, demonstrating that the DEDs of caspase-8 act to regulate not only cell death, but also differentiation and senescence.

## Results

### Caspase-8 Regulates Terminal Differentiation

**Figure 1:**
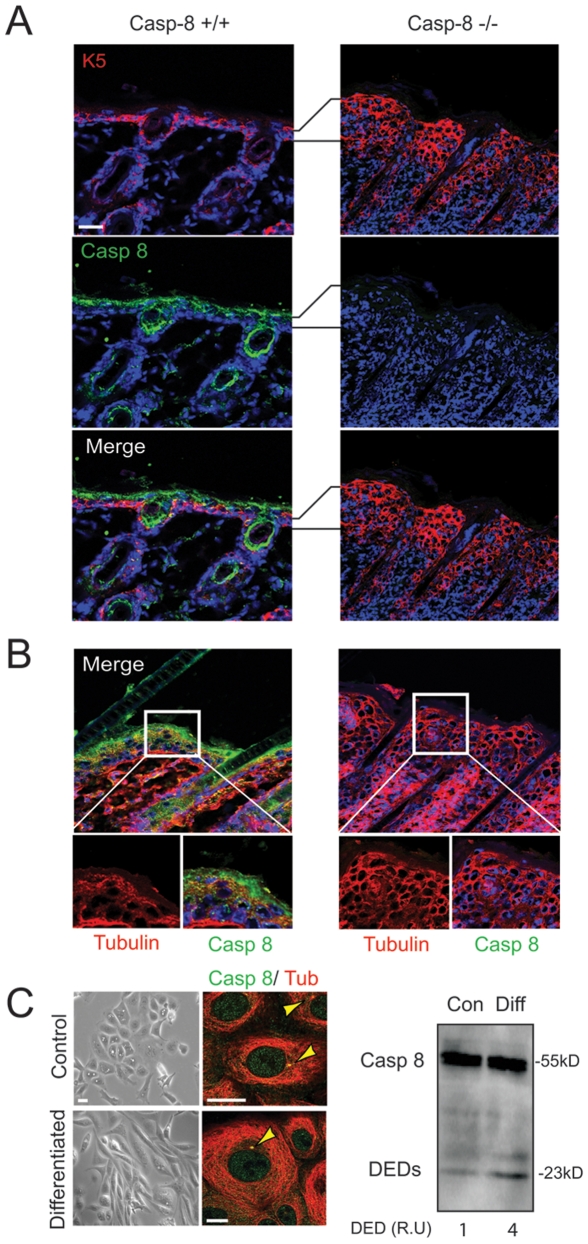
Effects of caspase-8 downregulation in skin maturation. **A.** Immunohistochemistry analysis performed in skin from wildtype and caspase-8 knock out mice. Caspase-8 DEDs are stained in green, keratin 5 in red and nuclei in blue. Brackets denote expanded epidermis in the caspase-8 knockout mice. **B.** Microtubule staining (red channel) in skin from wild type and caspase-8 knockout mice. Insets show magnified views of the microtubule patterning in wild type and caspase-8 knockout mice. **C.** **Left,** Confocal images of HaCat undifferentiated and differentiated cells stained for caspase-8 DEDs (green channel) and microtubules (red channel). Yellow arrows indicate centrosomes. **Right,** Immunoblot analysis of caspase-8 expression in undifferentiated or differentiated keratinocytes. Lysates were probed with antibody recognizing the DEDs of caspase-8.

Caspase-8 has been shown to play non-apoptotic roles in many tissues [1], [3], [20], [21]. Caspase-8 is particularly enriched in the skin [20], with a notable accumulation in the differentiating layers of the epidermis (Figure 1A, B and Figure S1A and B). Interestingly, the skin of caspase-8 knockout mice shows clear signs of hyperproliferation, particularly through the basal and spinous layers (Figure 1A). Examining the skin of K14^Cre^/C8^flox/flox^ animals, we found that loss of caspase-8 expression is associated with a deficient commitment of the basal cells to undergo terminal differentiation. In addition to epidermal thickening, the keratinocyte marker K5 is not restricted to a single cell layer, but rather is maintained throughout several cell layers (Figure 1A). Moreover, in caspase-8-deficient animals, the microtubules of the keratinocytes remained largely cytosolic rather than peripheral (Figure 1B), suggesting that the uncoupling of microtubules from the centrosome, associated with keratinocyte exit from proliferation, was disrupted [22]. Together, these results indicated that caspase-8 plays an important role in attenuating skin proliferation and/or modulating skin differentiation.

To examine this further, we next used a keratinocyte model of induced differentiation [23]–[25]. Differentiation of HaCat cells did not induce significant DEVDase (caspase) activity, nor did it promote accumulation of caspase-8 activation products such as the cleaved fragment p43 (Figure 1C and *data not shown*). The level of pro-caspase-8 expression did not change during this process, however, we did detect a modest accumulation of the DED domains among differentiating and non-proliferative cells (Figure 1C, right). We also detected the DEDs of caspase-8 at the centrosome, and this persisted during reorganization of the microtubules upon differentiation (Figure 1C, left).

**Figure 2:**
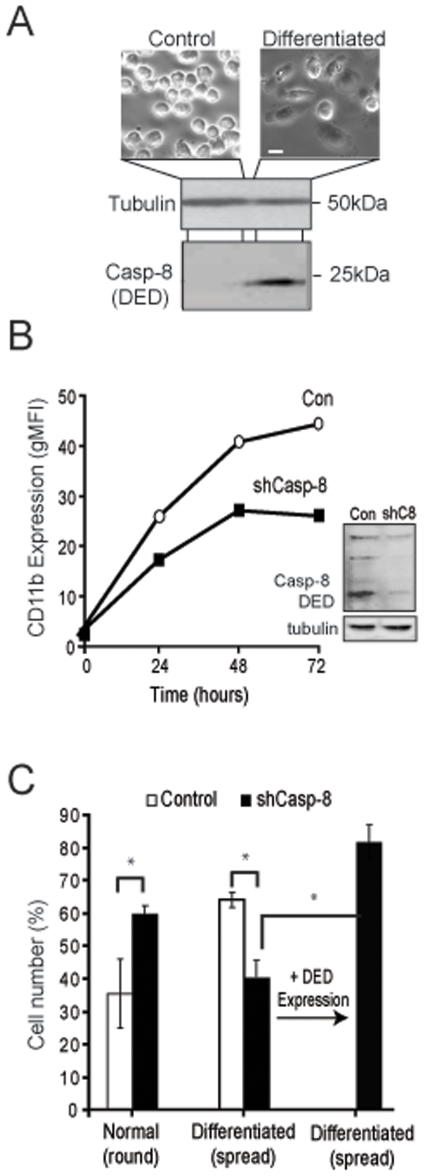
Caspase-8 DEDs are required for terminal differentiation of monocytes. **A.** Images of undifferentiated U937 monocytes (control) and differentiated macrophages (scale bar = 10 µm). Immunoblot analysis of caspase-8 expression among U937 undifferentiated monocytes (control) and differentiated macrophages. Lysates were probed with antibody recognizing the DEDs of caspase-8, with analysis of tubulin shown as a loading control. **B.** Monocytes were induced to differentiate by treatment with phorbol esters after infection with lentivirus encoding shRNA to caspase-8 or a control shRNA. Flow cytometry of control and caspase-8 knockdown U937 cells using CD11b expression as a reporter of differentiation. DED expression during differentiation was assessed by immunoblot analysis of these lysates with an antibody to caspase-8 DED domain (inset). **C.** Evaluation of U937 differentiation was performed by direct microscopic assessment using standard morphological criteria (round undifferentiated and spread differentiated). Data were analyzed with Fisher's Exact Test (*, significant difference, p≤0.05).

To evaluate whether the DEDs of caspase-8 influence cell differentiation, we next examined myeloid differentiation, in which caspase-8 has been implicated *in vivo* [3], [8]. Phorbol-ester-induced differentiation of U937 cells resulted in DED accumulation, as determined via immunoblot analysis with a monoclonal antibody to the DEDs of caspase-8 (Figure 2A). No caspase-8 DEDs were observed in differentiated U937 cells in which caspase-8 expression was silenced (U937-shC8) (Figure 2B, inset). Differentiation of the U937-shC8 cells was consistently compromised relative to control cells expressing caspase-8 (Figure 2B and C), both, by expression of the macrophage marker CD11b when scored by flow cytometry (Figure 2B) or by morphological criteria (Figure 2C). Importantly, re-expression of the DEDs was sufficient to rescue the differentiation process (Figure 2C). Together, these results support the notion that the DEDs of caspase-8 regulate terminal differentiation.

### Caspase-8 DED Suppress Tumor Growth

**Figure 3:**
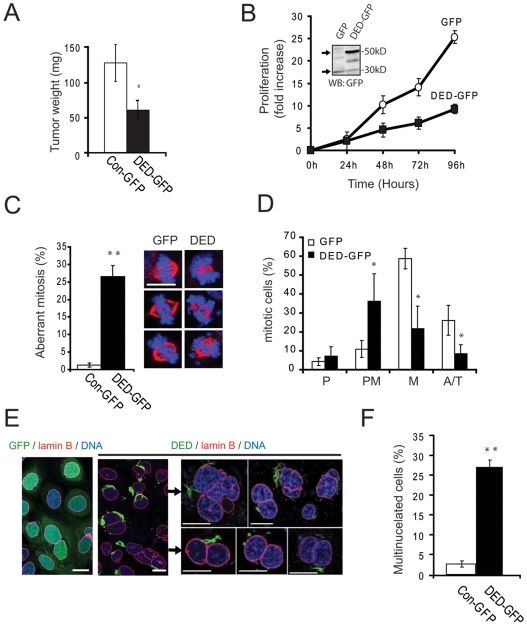
Caspase-8 DEDs decrease tumor burden, proliferation and induce multinucleation. **A.** Evaluation of tumor growth in chick embryos of NB7 neuroblastoma cells stably transfected with DED-GFP or control GFP. Data were analyzed with U-Mann-Whitney Test (*, significant differences, p≤0.05). **B.** The proliferation capacity of NB7-GFP or NB7 DED-GFP was monitored by direct counting of cells after 24, 48, 72 and 96 hours in culture after being seeded at 100, 000 cells/ml. **C.** Representative immunofluorescent images are shown, with alpha tubulin (red channel) and DNA/chromosomes (blue channel) evident in mitotic cells (scale bar = 10 µm). Microscopic quantification of aberrant mitosis. Data were analyzed with T-student Test (*, significant differences, p≤0.05; **, very significant differences, p≤0.01). **D.** Quantification of mitotic cells expressing GFP or DED-GFP. The distribution of mitotic cells is significantly different in these populations (P = Prophase, PM = Pro-metaphase, M = Metaphase and A/T = Anaphase/Telophase). Data were analyzed with T-student Test (*, significant differences, p≤0.05). **E.** Confocal microscopy indicating the presence of multinucleated cells within the DED-GFP cells, but not those expressing GFP (scale bar = 10 µm). **F**. Quantification of multinucleated NB7 GFP and NB7 DED-GFP cells.

Our observations suggested that the DEDs of caspase-8 were responsible for influencing terminal differentiation events. Neoplasms such as neuroblastoma can arise in part due to the failure of precursor cells (neuroblasts) to differentiate correctly [19]. Aggressive disease is associated with the loss of caspase-8 [26], while spontaneously resolving tumors (neuroblastoma type IV-S) will frequently express caspase-8 [27]. We confirmed that ectopic expression of DEDs among cells that already express caspase-8 induces apoptosis among caspase-8 positive cells [14], [28]. Many of these cells express readily detectable levels of DEDs, and appear to be intolerant to additional expression. Therefore, we next used caspase-8-deficient neuroblastoma cells to evaluate the role of caspase-8 DEDs in tumor progression. Interestingly, the growth of DED-GFP reconstituted neuroblastoma tumors in the chick chorioallantoic membrane was significantly inhibited relative to control tumors expressing GFP alone (Figure 3A). Accordingly, we found that the DED-GFP expressing cells did proliferate significantly less than control cells expressing GFP (Figure 3B). Thus, DED expression influenced neuroblastoma proliferation *in vitr*o and *in vivo*. The effect did not appear to be related to ”toxicity“ of DEDs, as the reconstituted neuroblastoma expressed similar levels of DEDs compared to other cell lines examined (COS-7, NB16; reference 14).

### DED Expression Causes Mitotic Defects

Interestingly, DED association with microtubule-containing structures can influence cell responses to paclitaxel (14), and thus, DED association with microtubules might influence cell division and/or proliferative arrest. The DEDs of caspase-8, but not c-FLIP, interact with stable microtubule structures (Figure S2), including centrosomes, spindle poles and midbodies in neuroblastoma cells [14] as well as keratinocytes and other epithelial cells (Figure S3A). Microtubule dynamics play a key role in regulating normal cell division, therefore, we tested the effect of DED expression on mitotic progression. Notably, we found an increased incidence of aberrant mitotic figures in DED-expressing neuroblastoma cells (Figure 3C). This was associated with an enrichment of DED-expressing cells in pre-metaphase relative to GFP-controls, suggesting mitotic delay (Figure 3D). In post-mitotic cells, DED-GFP expression was associated with multinucleation (Figure 3E, F, and Supplementary Figure S3B). Intriguingly, other cells which exhibit high basal levels of multinucleation, such as COS-7 [29] also expressed abundant caspase-8 DEDs (Figure S2C). Thus, the DEDs of caspase-8 influence normal cellular mitotic function, impacting cell proliferation.

In contrast, the decreased proliferation was not explainable simply by mitotic defects leading to apoptosis, since faster proliferation and larger tumors *in vivo* were derived from cells expressing a pro-apoptotic (wt) form of caspase-8. This caspase-8 GFP construct exhibited higher levels of apoptosis than the DED-GFP cells, but nonetheless grew larger *in vivo* and faster *in vitro* (Figure S4C, D and E). These studies also demonstrated that DEDs expressed as part of an inactive caspase-8 holoprotein (Casp8*-GFP) showed no deleterious effects on the cell (Figure S3C and E), possibly due to intra-domain interactions in the native procaspase. Moreover, DED expression in other caspase-8-deficient tumors (small cell lung carcinoma) [30], similarly impacted proliferation and resulted in multinucleation (Figure S4F), thereby validating the concept that DEDs contribute to mitotic crisis and cell cycle arrest.

### DEDs Activate the p53 Pathway and Induce Apoptosis or Cell Cycle Arrest

**Figure 4:**
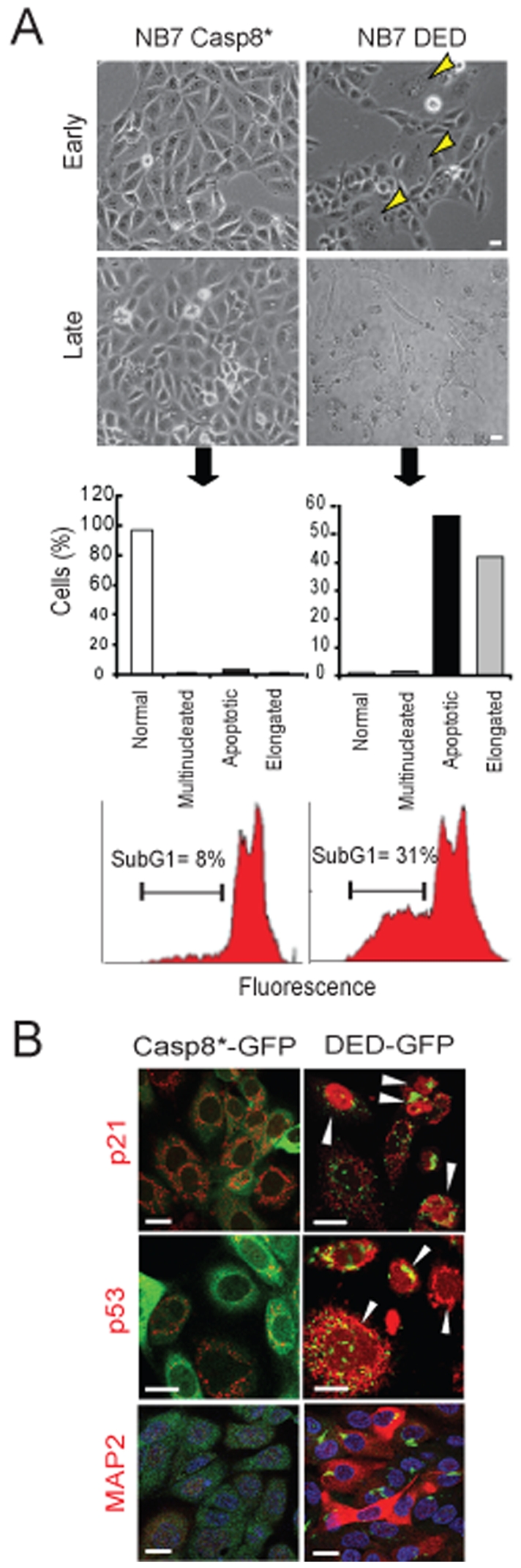
Caspase-8 DEDs activate p53, p21 and trigger apoptosis or cell cycle arrest. **A.** Bright field images of NB7 neuroblastoma cells expressing DED-GFP at passages 8 (early) and 30 (late) (scale bar = 10 µm). At earlier passages, a few multinucleated DED-expressing cells are detectable (yellow arrows). By passage 30, DED-GFP cells show high numbers of elongated or apoptotic cells. Late passage cells were stained with propidium iodide and assessed by flow cytometry to quantitate the sub-G1 apoptotic population at passage 30. **B.** Confocal microscopy confirmed the increase in nuclear expression of p21 and p53 (two markers of cell cycle arrest) and the cytoplasmic upregulation of the neuronal differentiation marker MAP2 in DED-GFP cells at passage 25 (scale bar = 10 µm). White arrows show nuclear accumulation of p21 and p53 (red channel).

We considered that selection pressure might result in the loss of caspase-8 DEDs, and increased proliferation and tumor formation might result with continued passage of the cells. To our surprise, we observed that proliferation of neuroblastoma cells did not increase, and that DED-expressing cultures arrested ∼30 passages after initial selection. Beyond early passages (p5–p12), multinucleation was increasingly common among the DED-expressing cells (Figure 4A upper panels). At later passages, cells either died or exhibited striking changes in phenotype, including elongate morphology and loss of proliferation (Figure 4A lower panels). Testing these cells for classical markers of cell cycle arrest [18], we observed an accumulation of both p21 and p53 in DED-GFP expressing cells (Figure 4B and Figure S4A, left panel). In agreement with our previous results in myeloid cells (Figure 2) and keratinocytes (Figure 1), this induced proliferative arrest that was accompanied by expression of neuronal differentiation markers such as MAP2 (Figure 4B) and βIII tubulin (Figure S5A, right panel). Therefore, the DEDs of caspase-8 appeared to influence both, multinucleation and the acquisition of cellular differentiation in the neuroblastoma cell model. To further validate a role of the DEDs in proliferative arrest, we used primary endothelial cell cultures, a known model of replicative senescence (Figure S5B and C). Endothelial cells also accumulated caspase-8 DEDs, and exhibited multinucleation at later passages (p6–p7) (Figure S5B). Knockdown of caspase-8 did not prevent senescence among the endothelial cells, but did reduce multinucleation and typically permitted additional 2–3 passages prior to arrest (Figure S5C). Together, the results support a model in which DED interaction with stable microtubule structures arrests proliferation and induces differentiation.

### DED Microtubule Binding Is Required for Cell Cycle Arrest and Differentiation

**Figure 5:**
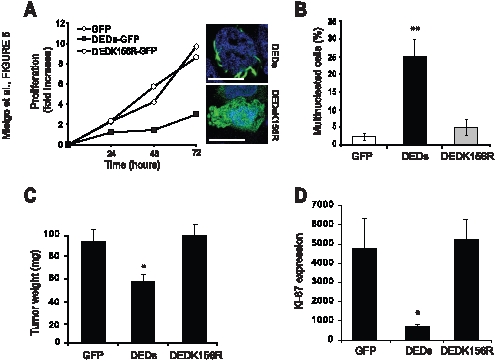
DED binding to microtubules and centrosomes via Lysine 156 is required for cell cycle arrest. **A.** The proliferation capacity of NB7-GFP, NB7 DED-GFP and NB7 DEDK156R-GFP was monitored by direct counting of cells after 24, 48 and 72 hours in culture after being seeded at 100 000 cells/ml. Values are given for one experiment representative of three. Confocal microscopy images show the localization of wildtype DED-GFP at microtubules and centrosomes and the cytoplasmic localization of the DEDK156R-GFP mutant (Insets). **B.** Quantification of multinucleated NB7 GFP, NB7 DED-GFP and NB7 DEDK156R-GFP cells. **C.** Tumor mass (wet weight) of tumors harvested from the chorioallantoic membrane of chicks Tumors were seeded at passage 15. The mass of the tumor cells at seeding was 40 µg. **D.** The relative proliferation of the tumors, assessed by Ki-67 expression within tumor sections, was assessed by immunofluorescence. The fluorescent pixels per low power field field is shown. Data were analyzed with T-student Test (*, significant differences, p≤0.05; ** p≤0.01).

Caspase-8 DEDs contain a microtubule-association motif (KLD) present in the second DED (DEDb) [14]. The motif is required for caspase-8 localization to centrosomes, microtubules, spindle poles and midbodies (Figure S2A)[14]. To test whether this microtubule binding function was required for the differentiation and anti-proliferative function of the DEDs, we next evaluated the proliferative capacity of NB7 cells expressing a mutant caspase-8 DED construct (K156R) that selectively lacks microtubule association, but still associates with other DED-containing proteins [14]. In contrast to neuroblastoma expressing wildtype DEDs, those expressing the K156R mutant were unaffected by DEDK156R expression. These cells did not differentiate, and proliferated similar to untransfected controls with no increased incidence of mitotic defects (Figure 5A and B). Accordingly, in tumors grown in the chick CAM, the DEDK156R cells grew similar to those expressing GFP (Figure 5C), while the expression of DEDs arrested growth, with a corresponding decrease in cell proliferation (Figure 5D). These results demonstrate that microtubule association is critical for DED-mediated differentiation and arrest of proliferation. Together, these data reveal a new role for caspase-8 DEDs in the regulation of cell differentiation and senescence.

## Discussion

In this study, we characterize a new function for the death effector domains of caspase-8 in cell cycle regulation. First, we find that caspase-8 DEDs accumulate in cells undergoing terminal differentiation. Silencing of caspase-8 disrupts or delays differentiation, while reintroduction of DEDs restores differentiation potential. We further report that DED expression is sufficient to impair tumor growth and cell proliferation, promoting mitotic defects that foster cell death or cell cycle arrest and terminal differentiation. Finally, we demonstrate that these events require a critical lysine (K156) in a microtubule binding motif in the second DED (DED-b). These observations together suggest that caspase-8 DEDs function as a tumor suppressor, acting as an anti-proliferation and differentiation-inducing element. It is tempting to speculate that this type of mechanism may act in some spontaneously regressing tumors, such as stage IV-S neuroblastoma, however, such a mechanism would be co-dependent upon other classic tumor suppression pathways, including p53 and p21. Interestingly, the cell lines which can maintain higher levels of DEDs, such as COS-7, also have documented defects in p53 signaling.

Reconstitution of DED expression in the caspase-8 deficient NB7 cells similarly leads to an increase in multi-nucleation. This effect resembles the effect of microtubule-directed agents which disrupt mitotic spindle formation and chromosome segregation. However, these microtubule disrupting agents do not efficiently induce differentiation, suggesting that the DEDs may play additional roles following microtubule binding. Collaborative effect is observed between caspase-8 DEDs and microtubule stabilizing agents, but not microtubule-disrupting drugs [14]. It is likely that this occurs because microtubule-stabilizing agents act to increase DED association with microtubules [14].

Death domain proteins are a large and evolutionarily ancient family. Defined by Tube and Pelle, each domain consists of six peptide helices, with the specific homology placing family members into subfamilies that include the DEDs, the classic death domain (DD) proteins, the pyrin domains proteins, and the caspase recruitment domain (CARD) families [31]. Interactions among these proteins are frequently homotypic or within a subfamily, but interactions between different families, and with unrelated proteins, are unknown. Microtubules have several surface helices with which microtubule-binding proteins interact, as well as cell surface clefts bound by KLD/KID-containing proteins such as kinesins, Tau, and MAP2C [32], [33]. While other DED proteins lack the KLD motif in the turn between helix 4 and 5 of the DED structure, it remains possible that some might still associate with microtubules. For example, at least one death domain protein with a CARD fold, CARD6, interacts with microtubules [34].

It is also possible that DED-mediated effects can influence differentiation or proliferation arrest among cells in which the apoptotic pathway is compromised. For example, it is common for tumor cells to express high levels of anti-apoptotic proteins that interrupt the catalytic cascade [35], or which otherwise alter the threshold of caspase-8 activation required for apoptosis [36], [37]. Alternatively, low level activation of caspase-8 may contribute to aberrant cell growth, since activation of the extrinsic death pathway at levels which do not induce apoptosis can foster NF-κB signaling [38]. Since caspase-8 maturation occurs concomitant with the accumulation of DEDs, DED accumulation may act to oppose NF-κB signaling, functioning as a tumor suppressor via their microtubule-associated function. Given the caveats of the studies performed here, it would appear that the DEDs have a better stability than the catalytic domain, which is a target of RING protein-mediated clearance [37].

In summary, we describe a novel function for caspase-8 as an orchestrator of not only apoptosis but also differentiation and senescence. The function is unexpectedly associated with the amino-terminal death effector domains, and does not require caspase-8 catalytic activity. The ermerging roles of this multifunctional protein, and its surprising interactions at the nexus of the cellular migration, proliferation, differentiation and apoptosis pathways, continue to offer key insights into the signaling cross-talk that regulates cell fate.

## Methods

### Cell Culture and Differentiation

Human leukemic cell line U937 (CRL-1593.2, American Tissue Culture Collection, Rockville, MD) were grown in RPMI 1640 medium with glutamax-I (Gibco, Invitrogen, Carlsbad, CA) supplemented with 10% fetal bovine serum (HyClone, Logan, UT) under standard cell culture conditions.

Differentiation was induced by culturing cells in the presence of 20 nM Phorbol 12-myristate 13-acetate (Sigma-Aldrich, St. Louis, MO) during indicated time.

After treatment, transmission light pictures were taken with an inverted Microscope (Eclipse, Nikon Inc, USA) using MetaMorph software (Molecular Devices, Sunnyvale, CA). Adherent cells were harvested by first incubating the cells with Versene, a non enzymatic dissociation buffer (Gibco, Carlsbad, CA) for 5 min, followed by collecting the cells using a cell scraper. Cellular differentiation was quantified by flow cytometry. Briefly, cells were resuspended in PBS + 5% FBS, human IgG (1∶100) was added and cells were incubated on ice during 45 min to block unspecific binding. Cells were stained using an anti human CD11b-APC antibody (M1/70, Miltenyi Biotech Auburn, CA).

### Viability Cell Cycle Analysis

Cell viability was analyzed by flow cytometry following propidium iodide (PI) staining, essentially as described before [34]. Briefly, cells were cultured for 48 hours and harvested, resuspended in ice cold PBS containing 10 µg/ml of Propidium iodide (PI) and analyzed by flow cytometry. The extent of apoptosis was determined by plotting PI fluorescence versus the forward scatter parameter, using the Cell Quest program. For DNA content analysis (cell cycle distribution), cells were previously permeabilized in methanol at −20°C, for 15 minutes and resuspended in PBS containing RNase A and 10 µg/ml of PI. Samples containing roughly 2×10^4^ cells were analyzed using the Cell Quest program.

### Immunoprecipitation and Immunoblotting

Cells were lysed in either NP40 lysis buffer (150 mM NaCl, 50 mM Tris Base pH 7.4, 1% NP40) or RIPA buffer (50 mM Tris pH 7.4, 100 mM NaCl, 0.1% SDS) supplemented with complete protease inhibitor mixture (Roche), 50 mM NaF and 1 mM Na_3_VO_4_ and centrifuged at 13,000 g for 10 min at 4°C. Protein concentration was determined by BCA assay. For immunoprecipitation, 500 µg of protein was incubated with 2 µg of rabbit anti-GFP antibody (Abcam) overnight, at 4°C. Complexes were precipitated with 25 µl of protein A/G (Pierce). Beads were washed five times, eluted in boiling Laemmli buffer, resolved on 10% SDS-PAGE and immunoblotting was performed with mouse anti-alpha tubulin antibody (1∶1000). For immunoblot analysis, 30 µg of protein was boiled in Laemmli buffer and resolved on 10% gel.

### Cell Culture and Transfections

Human neuroblastoma cells (NB7), deficient in caspase-8 were cultured in RPMI supplemented with 10% fetal bovine serum, glutamine and non-essential aminoacids. Human keratinocytes (HaCat), human epithelial cells (A549) and monkey fibroblasts (COS-7), were cultured in DMEM, supplemented as previously described. Human endothelial cells (HUVECs) were cultured in M199 with endothelial cell supplement, 10% fetal bovine serum, glutamine and minimal essential aminoacids. NB7 cells deficient in caspase-8 were transfected using the Fugene reagent following manufacturer's protocol. Stable cell lines were selected with 500 µg/ml G418 (Gibco) and sorted by Flow cytometry for GFP positive cells. Caspase-8 expression was confirmed by immunoblotting with caspase-8 N-terminus (BD Pharmingen) and C-terminus antibodies (hybridomas, clones C5 and C15, were a gift from M. Peter, University of Chicago, USA). To create caspase-8 deficient and control cell lines, U937 and HUVECs cells were infected with lentivirus encoding shRNA to caspase-8 (Open Biosystems) or a control shRNA as described previously (Wrasidlo et al., 2008).

### Vectors and Constructs

DNA for caspase-8 was kindly provided by Guy Salvesen, Burnham Institute, La Jolla, USA. Full length caspase-8-GFP (C8-GFP) and inactive mutant C360A (C8*-GFP) fusion proteins were cloned into C1pEGFP (Clontech Laboratories). Death effector domain from caspase-8 (DED-GFP), DED1-GFP and DED2-GFP fusion proteins were generated using 5′ and 3′ primers containing unique HindIII and BamHI restriction sites respectively, and cloned into N2pEGFP. K156R, S109A and D135A mutations were introduced in DED using the QuikChange Mutagenesis kit (Stratagene) with appropriate mutagenesis primers. DED myc-His fusion protein was made with the same primers but cloned into pcDNA3.1 myc-His (Invitrogen).

### Tumor Growth

For avian tumor studies, 5×10^6^ neuroblastoma cells suspended in 40 µl of complete medium were seeded on 11-day-old chick chorioallantoic membrane [35]. Tumors were left to develop for 7 days and were then resected and weighed.

### Immunocytochemistry, Immunohistochemistry and Confocal Microscopy

Cells were permitted to attach to coverslips, fixed with 4% PFA and permeabilized in PBS containing 0.1% triton for three minutes, blocked for 60 minutes, at room temperature with 2% BSA in PBS. Cell were stained with monoclonal antibody to amino terminus death effector domain of caspase-8 (Pharmingen and Calbiochem), p53 (Cell signaling), p21 (Santa Cruz) polyclonal antibody specific for alpha tubulin (Abcam), gamma tubulin (Abcam), pericentrin (Abcam) or lamin B (Santa Cruz). All primary antibodies were used at 1∶100 dilution, for two hours at room temperature. After washing several times with PBS, cells were stained for two hours at room temperature, with secondary antibody fluorescently labelled in green or red, specific for mouse, rabbit or goat (Invitrogen) and diluted 1∶300. In some cases, cells were co-incubated with the blue DNA dye TO-PRO-3 (1∶1000) (Invitrogen).

Mouse skin isolated from P1 and P10 wildtype and knockout animals were frozen in OCT (Tissue-Tek). Caspase-8, α tubulin and epidermal differentiation markers, K5, K1 and loricrin were stained in 8 µm frozen sections after tissues were fixed for 10 min in cold acetone or cold acetone/methanol (for α tubulin staining). For nuclear staining, TO-PRO-3 (invitrogen) was added to the secondary antibody dilution. Immunofluorescence was detected using Alexa-fluor 488 and Alexa-fluor 568 secondary antibodies (Invitrogen). Samples were mounted in Vectashield hard set mounting media (Vector Laboratories) and imaged on a Nikon Eclipse C1 confocal microscope.

## Supporting Information

Figure S1 Expression of caspase-8 DEDs in the differentiated layers of the epidermis. A. Immunohistochemistry experiments performed in mouse skin confirm expression of caspase-8 DEDs (green channel) in the differentiated layers of the skin (spinous, granular and cornified layers). The basal layer is stained in red with keratin 5 and nuclei of keratinocytes are stained with TO-PRO-3 (blue channel). Insets show magnified views of the skin. B. Immunohistochemistry experiments performed in sequential sections of mouse skin confirm expression of caspase-8 DEDs (green channel) in the differentiated layers of the skin (spinous, granular and cornified layers). The spinous and granular layers are stained in red with keratin 1 and loricrin, respectively, and nuclei of keratinocytes are stained in blue.
(5.29 MB TIF)

Figure S2 Assessment of the localization of the DEDs from c-FLIP. The DEDs of c-FLIP and caspase-8 can bind several common targets. The distribution of GFP-fusion proteins containing the amino-terminal DEDs of c-FLIP and caspase-8B were compared. A. An alignment of the amino terminal region of caspase-8, containing the DEDs of caspase-8 and c-FLIP, is shown. The RxDLL motif (red) and a KLD motif (green) are shown. B. Immunofluorescence imaging of DED-GFP fusion protein localization (green channel) within NB7 cells. Nuclei are stained with TO-PRO (blue channel) and microtubules with anti-α tubulin (red channel) (Bar, 10 µm).
(14.21 MB TIF)

Figure S3 Endogenous DEDs associate with microtubules, centrosomes, spindle poles and midbodies and accumulate in multinucleated cells. A. Confocal microscopy images of COS-7, HeLa, HaCat and NB16 cells showing localization of caspase 8 DEDs (green channel) at the microtubules, centrosomes, spindle poles and midbody (red channel). Microtubules and midbodies are stained with {small case alpha}-tubulin, centrosomes and spindle poles are stained with anti-γ-Tubulin (red channel), the DEDs with amino-terminal caspase-8 antibody (green channel), and DNA/chromosomes with TO-PRO(blue channel). Centrosomes are imaged using a minimal confocal pinhole and fluorescence thresholding of 80% (scale bar = 10 microns). B. GFP and DED-GFP expressing cells were stained with propidium iodide and percentage of multinucleated cells (≥4N) was measured by flow cytometry. C. Confocal images showing COS-7 giant multinucleated cells (scale bar = 10 µ). Quantification of COS-7 and NB7 multinucleated cells. Immunoblot analysis showing expression of endogenous caspase-8 DED in COS-7 cells (inset). Data were analyzed with U-Mann-Whitney Test (significant differences, *, p<0.05; **, p<0.01).
(18.77 MB TIF)

Figure S4 DEDs impair tumor growth, proliferation and trigger defects in mitosis. A. Scheme of GFP-tagged caspase-8 derivatives, which express either the DEDs alone, the catalytic domain active or inactive (C360A), the holoenzyme (caspase-8B) active or inactive (C360A). B. Immunoblot analysis showing expression of GFP, caspase-8 inactive-GFP (C8*-GFP), caspase-8-GFP (C8-GFP), catalytic domain-GFP (CAT-GFP), catalytic domain inactive-GFP (CAT*-GFP) and DED-GFP. Upper panel: Western blotting with a C8 DEDs-specific antibody. Middle panel: Western blotting with a C8 Catalytic domain-specific antibody. Lower panel: Western blot with a tubulin-specific antibody, used as loading control. C. Proliferation assay performed with GFP, C8-GFP, C8*-GFP, CAT-GFP, CAT*-GFP and DED-GFP expressing cells. D. GFP, C8-GFP and DED-GFP expressing cells were stained with propidium iodide and percentage of apoptotic cells was measured by flow cytometry. Data were analyzed with U-Mann-Whitney Test (*, significant differences, p≤0.05; **, very significant differences, p≤0.01). E. Evaluation of tumor growth in chick embryos of NB7 neuroblastoma cells stably transfected with DED-GFP, Casp8-GFP, Casp8*-GFP or control GFP. F. Immunofluorescence images of human Small Cell Lung Carcinoma Cells (SCLC) transfected with DED-GFP or control GFP and Neuroblastoma NB7 cells transfected with DED-GFP, stained with lamin B (red channel) and a DNA dye (blue channel) show accumulation of micronuclei. White arrows show micronuclei or amorphous nuclei (scale bar = 10 µm).
(25.72 MB TIF)

Figure S5 Implication of caspase-8 DEDs in cell differentiation and senescence. A. Immunoblot analysis confirmed the increase of the cell cycle arrest marker p21 and the neuronal differentiation marker tubulin beta III in DED-GFP cells at passage 25. Immunoblot analysis of actin is shown as loading control. B. Bright field and confocal microscopy images of Human Umbilical Vein Endothelial Cells (HUVECs) at early passages (up to P4) and late passages (P6-P8). Nuclear p53 staining (red channel) confirms accumulation of senescent cells at late passages. Lamin B staining (red channel) shows accumulation of binucleated cells at late passages (yellow arrows) (scale bar = 10 µm). Immunoblot analysis with a caspase-8 DEDs specific antibody on HUVECs shows accumulation of DED at late passages. Immunoblot analysis of tubulin is shown as loading control. Quantification of multinucleated HUVECs at early and late passages. C. Quantification of multinucleation in wild type and lentivirus encoding shRNA to caspase-8 infected endothelial cells, at early and late passages. Immunoblot analysis showing caspase-8 expression in these cells (inset). Data were analyzed with U-Mann-Whitney Test (significant differences, *, p≤0.05; **, p≤0.01).
(21.02 MB TIF)
