# Supplementary figures and images for: The Death Effector Domains of Caspase-8 Induce Terminal Differentiation
Source: PLoS One. 2009 Nov 18;4(11):e7879. doi: 10.1371/journal.pone.0007879 (PMC2774162; doi:10.1371/journal.pone.0007879)

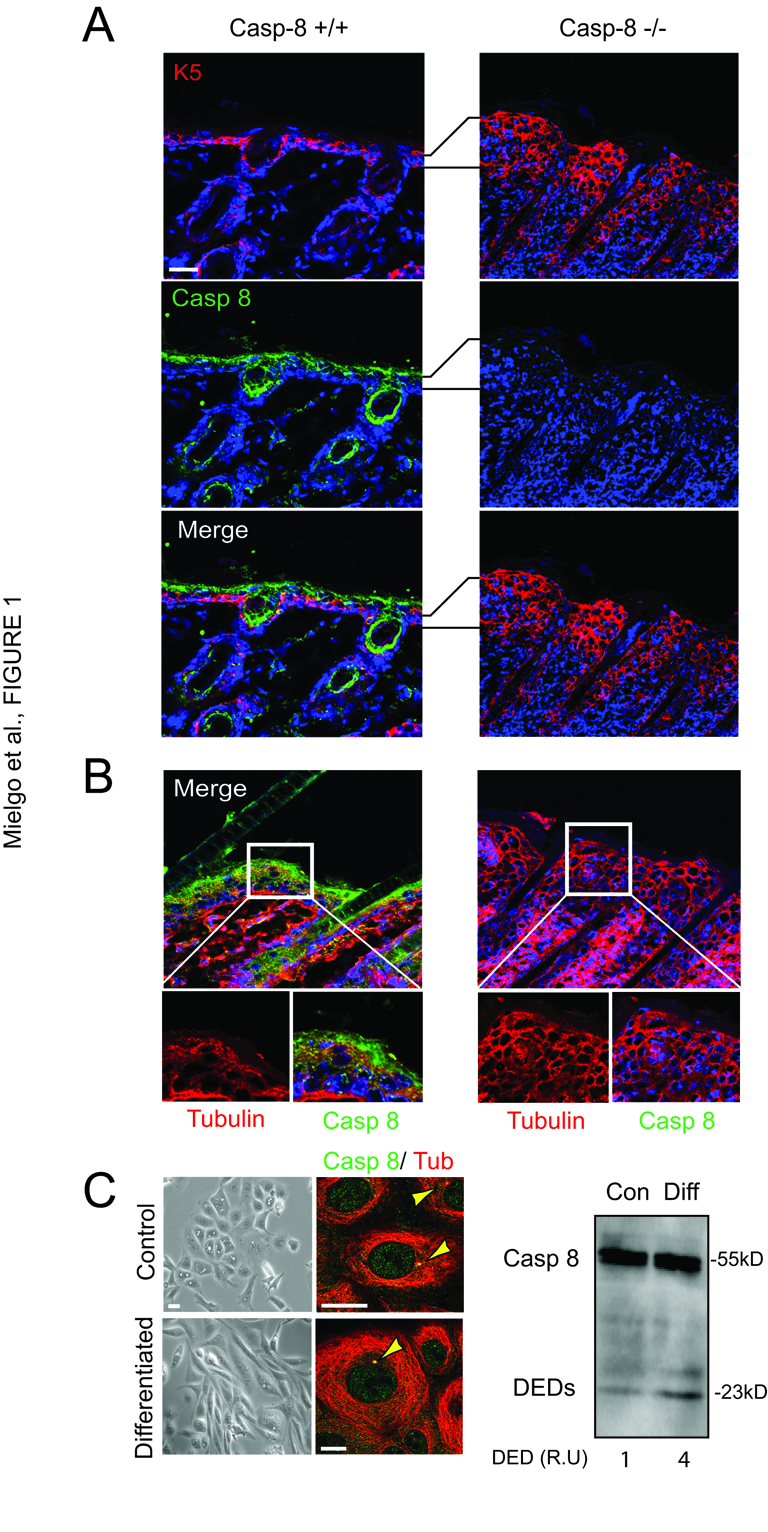

Supplement: Figure S1 — Expression of caspase-8 DEDs in the differentiated layers of the epidermis. A. Immunohistochemistry experiments performed in mouse skin confirm expression of caspase-8 DEDs (green channel) in the differentiated layers of the skin (spinous, granular and cornified layers). The basal layer is stained in red with keratin 5 and nuclei of keratinocytes are stained with TO-PRO-3 (blue channel). Insets show magnified views of the skin. B. Immunohistochemistry experiments performed in sequential sections of mouse skin confirm expression of caspase-8 DEDs (green channel) in the differentiated layers of the skin (spinous, granular and cornified layers). The spinous and granular layers are stained in red with keratin 1 and loricrin, respectively, and nuclei of keratinocytes are stained in blue. (5.29 MB TIF) [file pone.0007879.s001.tif]

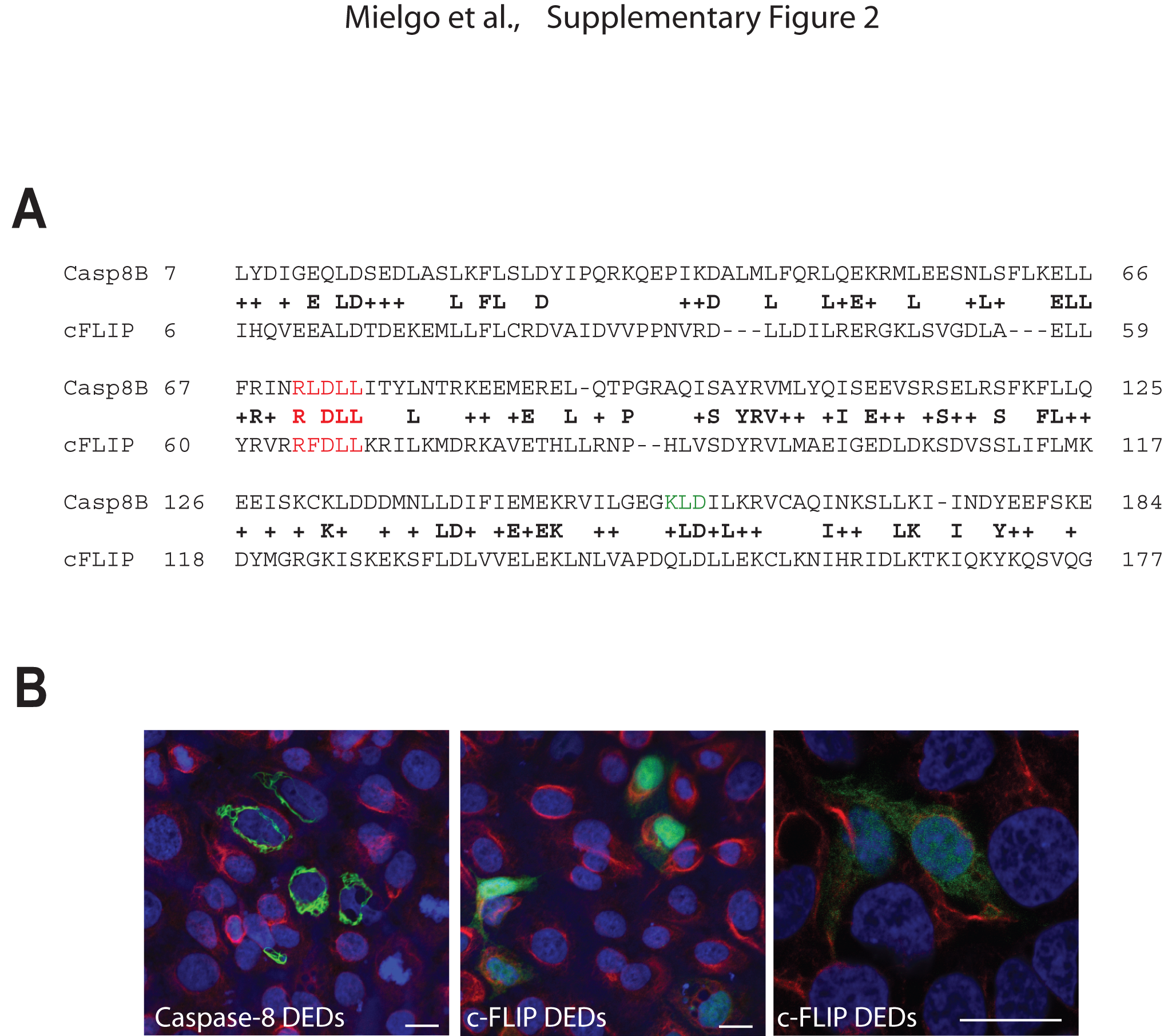

Supplement: Figure S2 — Assessment of the localization of the DEDs from c-FLIP. The DEDs of c-FLIP and caspase-8 can bind several common targets. The distribution of GFP-fusion proteins containing the amino-terminal DEDs of c-FLIP and caspase-8B were compared. A. An alignment of the amino terminal region of caspase-8, containing the DEDs of caspase-8 and c-FLIP, is shown. The RxDLL motif (red) and a KLD motif (green) are shown. B. Immunofluorescence imaging of DED-GFP fusion protein localization (green channel) within NB7 cells. Nuclei are stained with TO-PRO (blue channel) and microtubules with anti-α tubulin (red channel) (Bar, 10 µm). (14.21 MB TIF) [file pone.0007879.s002.tif]

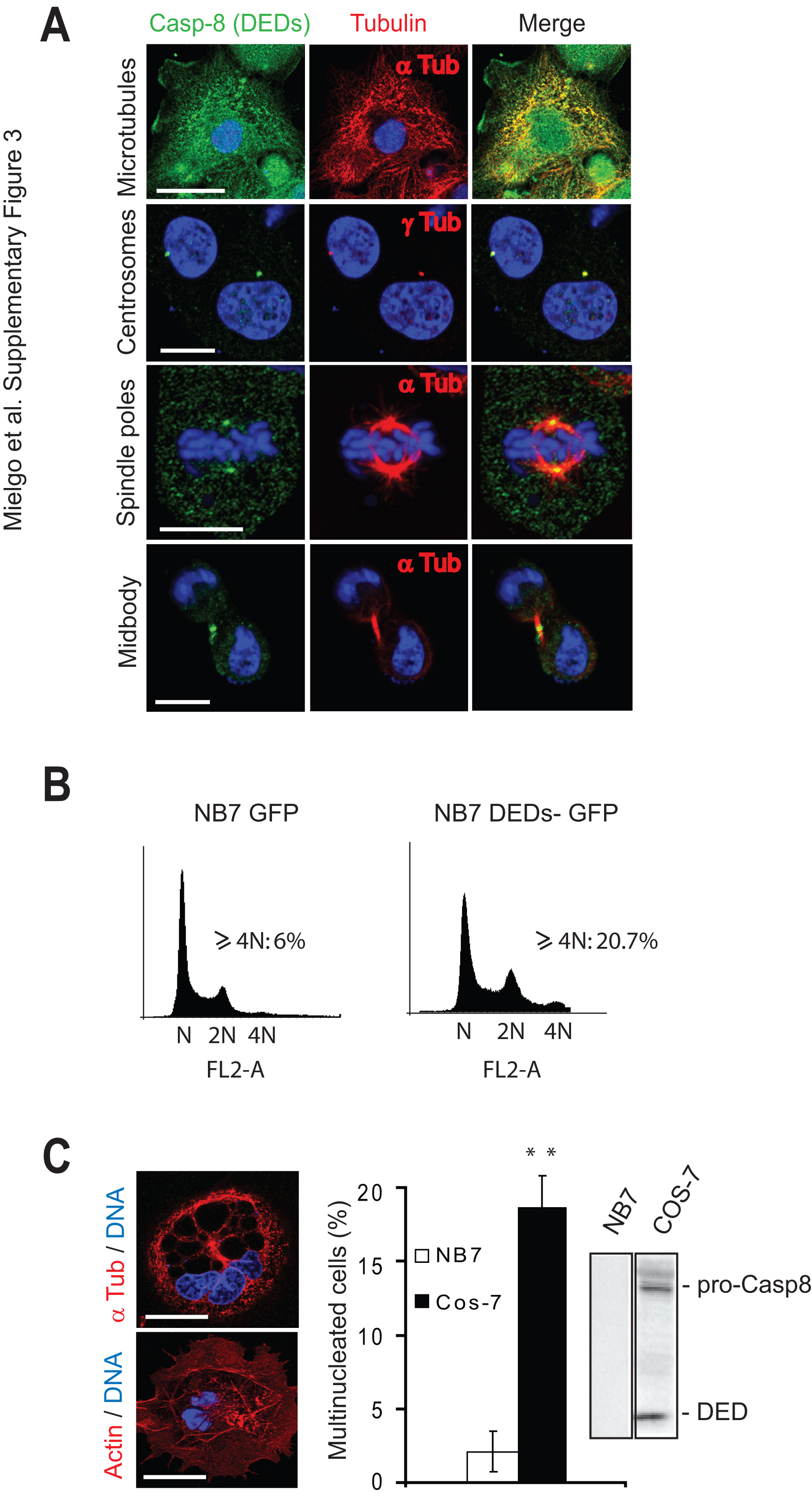

Supplement: Figure S3 — Endogenous DEDs associate with microtubules, centrosomes, spindle poles and midbodies and accumulate in multinucleated cells. A. Confocal microscopy images of COS-7, HeLa, HaCat and NB16 cells showing localization of caspase 8 DEDs (green channel) at the microtubules, centrosomes, spindle poles and midbody (red channel). Microtubules and midbodies are stained with {small case alpha}-tubulin, centrosomes and spindle poles are stained with anti-γ-Tubulin (red channel), the DEDs with amino-terminal caspase-8 antibody (green channel), and DNA/chromosomes with TO-PRO(blue channel). Centrosomes are imaged using a minimal confocal pinhole and fluorescence thresholding of 80% (scale bar = 10 microns). B. GFP and DED-GFP expressing cells were stained with propidium iodide and percentage of multinucleated cells (≥4N) was measured by flow cytometry. C. Confocal images showing COS-7 giant multinucleated cells (scale bar = 10 µ). Quantification of COS-7 and NB7 multinucleated cells. Immunoblot analysis showing expression of endogenous caspase-8 DED in COS-7 cells (inset). Data were analyzed with U-Mann-Whitney Test (significant differences, *, p<0.05; **, p<0.01). (18.77 MB TIF) [file pone.0007879.s003.tif]

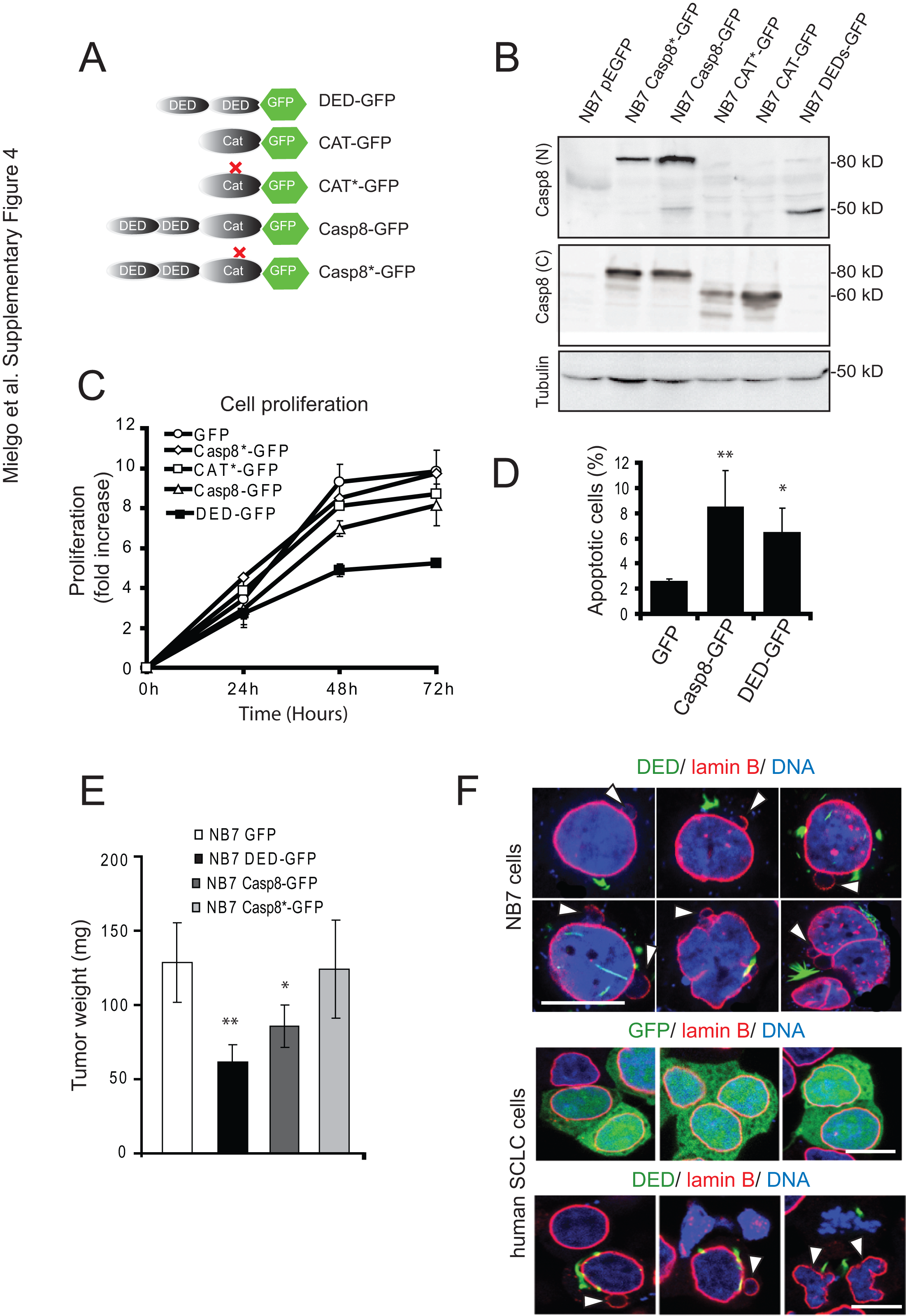

Supplement: Figure S4 — DEDs impair tumor growth, proliferation and trigger defects in mitosis. A. Scheme of GFP-tagged caspase-8 derivatives, which express either the DEDs alone, the catalytic domain active or inactive (C360A), the holoenzyme (caspase-8B) active or inactive (C360A). B. Immunoblot analysis showing expression of GFP, caspase-8 inactive-GFP (C8*-GFP), caspase-8-GFP (C8-GFP), catalytic domain-GFP (CAT-GFP), catalytic domain inactive-GFP (CAT*-GFP) and DED-GFP. Upper panel: Western blotting with a C8 DEDs-specific antibody. Middle panel: Western blotting with a C8 Catalytic domain-specific antibody. Lower panel: Western blot with a tubulin-specific antibody, used as loading control. C. Proliferation assay performed with GFP, C8-GFP, C8*-GFP, CAT-GFP, CAT*-GFP and DED-GFP expressing cells. D. GFP, C8-GFP and DED-GFP expressing cells were stained with propidium iodide and percentage of apoptotic cells was measured by flow cytometry. Data were analyzed with U-Mann-Whitney Test (*, significant differences, p≤0.05; **, very significant differences, p≤0.01). E. Evaluation of tumor growth in chick embryos of NB7 neuroblastoma cells stably transfected with DED-GFP, Casp8-GFP, Casp8*-GFP or control GFP. F. Immunofluorescence images of human Small Cell Lung Carcinoma Cells (SCLC) transfected with DED-GFP or control GFP and Neuroblastoma NB7 cells transfected with DED-GFP, stained with lamin B (red channel) and a DNA dye (blue channel) show accumulation of micronuclei. White arrows show micronuclei or amorphous nuclei (scale bar = 10 µm). (25.72 MB TIF) [file pone.0007879.s004.tif]

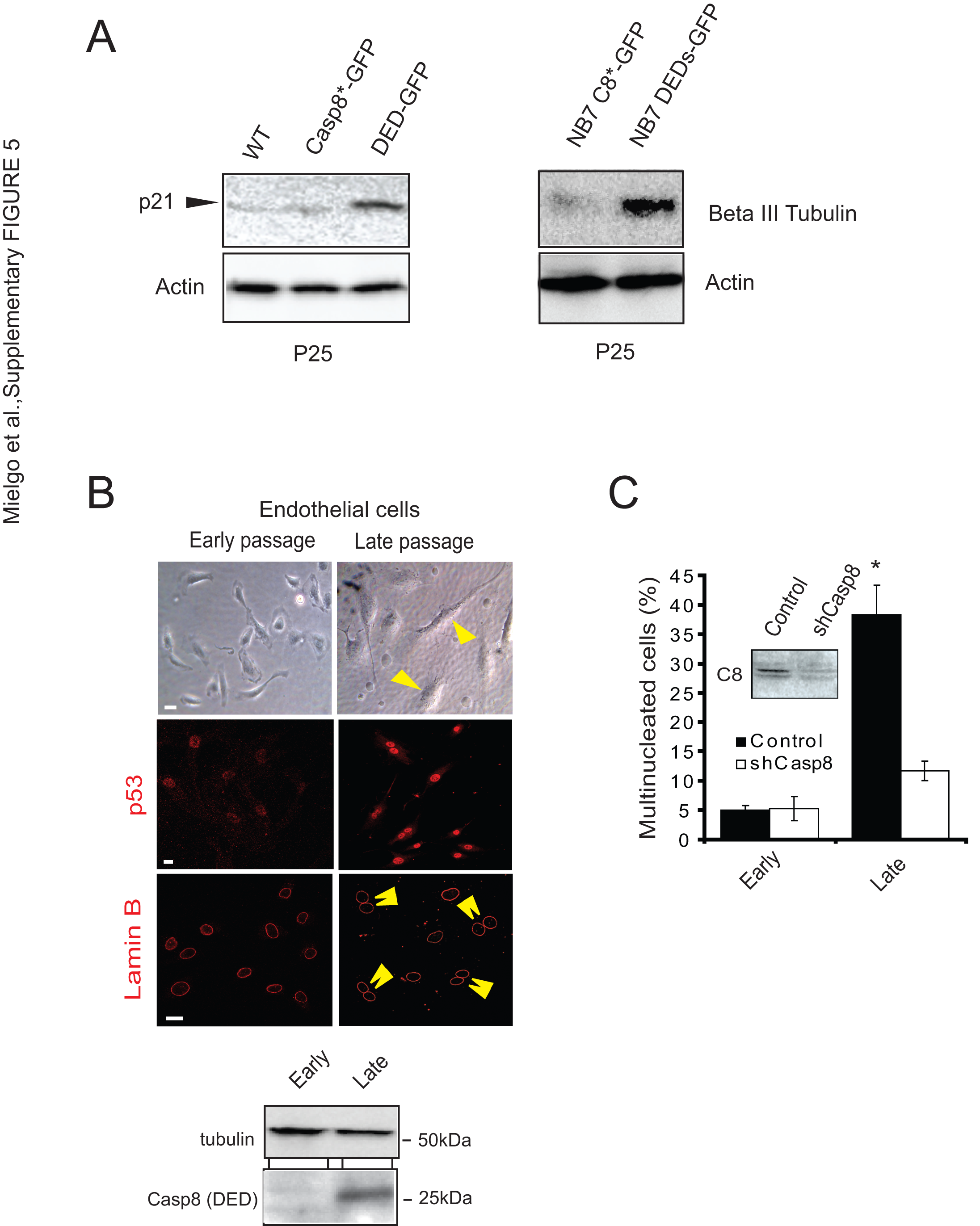

Supplement: Figure S5 — Implication of caspase-8 DEDs in cell differentiation and senescence. A. Immunoblot analysis confirmed the increase of the cell cycle arrest marker p21 and the neuronal differentiation marker tubulin beta III in DED-GFP cells at passage 25. Immunoblot analysis of actin is shown as loading control. B. Bright field and confocal microscopy images of Human Umbilical Vein Endothelial Cells (HUVECs) at early passages (up to P4) and late passages (P6-P8). Nuclear p53 staining (red channel) confirms accumulation of senescent cells at late passages. Lamin B staining (red channel) shows accumulation of binucleated cells at late passages (yellow arrows) (scale bar = 10 µm). Immunoblot analysis with a caspase-8 DEDs specific antibody on HUVECs shows accumulation of DED at late passages. Immunoblot analysis of tubulin is shown as loading control. Quantification of multinucleated HUVECs at early and late passages. C. Quantification of multinucleation in wild type and lentivirus encoding shRNA to caspase-8 infected endothelial cells, at early and late passages. Immunoblot analysis showing caspase-8 expression in these cells (inset). Data were analyzed with U-Mann-Whitney Test (significant differences, *, p≤0.05; **, p≤0.01). (21.02 MB TIF) [file pone.0007879.s005.tif]
